# Cellular and Molecular Targets of Resveratrol on Lymphoma and Leukemia Cells

**DOI:** 10.3390/molecules22060885

**Published:** 2017-05-27

**Authors:** Raffaele Frazzi, Manuela Guardi

**Affiliations:** Laboratory of Translational Research, Arcispedale S. Maria Nuova IRCCS, Viale Risorgimento 80, 42124 Reggio Emilia, Italy; manuela.guardi@asmn.re.it

**Keywords:** resveratrol, lymphoma, leukemia, molecular target

## Abstract

Resveratrol (RSV) is a well known chemopreventive molecule featuring anti-cancer properties. Our paper describes the main molecular targets of RSV linked to its antiproliferative activity on lymphoma and leukemia experimental models. It discusses further the most recent and most promising among these molecular targets for a translational application.

## 1. Introduction

Resveratrol (RSV, trihydroxystilbene) is a nonflavonoid plant polyphenol characterized by many beneficial properties for human health [1]. It has been known for a long time as an anti-inflammatory, anti-oxidant, chemopreventive, glucose-lowering and anti-aging molecule [2]. These several qualities have been investigated by many researchers over the years. In this review, we will focus on the anti-cancer properties of RSV directed towards lymphoma and leukemia. Specifically, the aim is to review the main molecular targets known to be hit, directly or indirectly, during the action of RSV on these hematologic malignancies. As an anticancer agent, RSV has pleiotropic effects, altering many different signaling pathways, leading to suppression of tumor cell proliferation, adhesion, invasion and metastasis, reduced signs of inflammation, angiogenesis and induction of apoptosis and differentiation [3,4,5,6].

RSV has been demonstrated to interact with a variety of molecular targets associated with cell cycle progression, apoptosis and survival pathways, tumor invasion and angiogenesis, cyclooxygenase-2, transcription factors, lysosomal cathepsin, Adenosine Monophosphate (AMP)-Activated Protein Kinase and reactive oxygen species (ROS) generation [7].

The information currently available concerning the antitumor activity towards lymphoma and leukemia need to be coordinated and integrated to effectively assess whether the properties of RSV are useful in a translational perspective.

## 2. Molecular Targets during Apoptosis Onset in Lymphomas

It has long been known that RSV can trigger apoptosis in several types of cancer cells like squamous cell carcinoma, lymphoma, leukemia, colon, breast, and others [8,9,10,11,12,13,14].

Indeed, several pathways are involved during RSV-mediated apoptosis [11,15]. Apoptosis is a physiological process resulting in a highly-regulated, programmed form of cell death that is a normal part of growth and development in multicellular organisms. Chemical compounds that affect apoptotic pathways in order to eliminate cancer cells are considered promising anticancer drugs.

**Figure 1:**
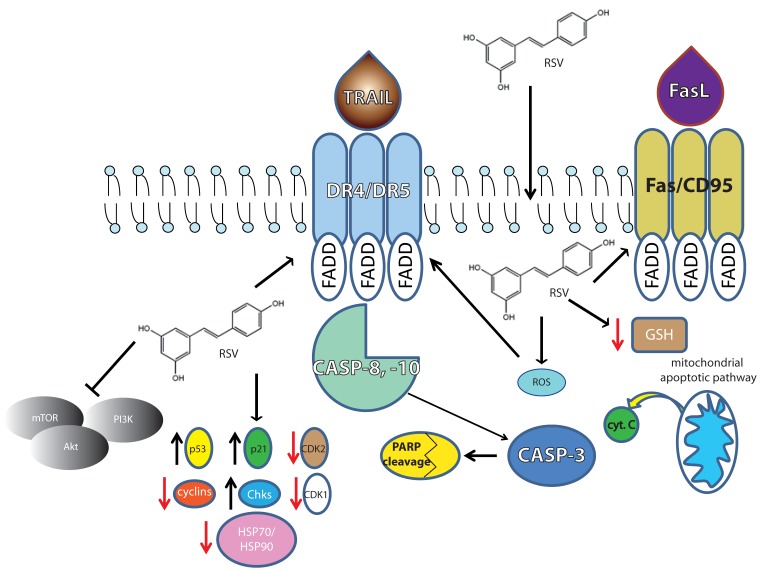
Representation of the main molecular targets demonstrated in lymphoma cells. The blunt-ended arrows represent a downregulating effect. Black, up-oriented arrows indicate up-regulation. Red, down-oriented arrows indicate downregulation.

Here, we try to underline the most representative pathways described for lymphoma and leukemia thus far (summarized in Figure 1 and Table 1).

### 2.1. The Intrinsic Apoptotic Pathway

Pterostilbene (a natural di-methylated analog of RSV) activates the intrinsic apoptotic pathway in diffuse large B-cell lymphoma cell lines (DLBCL) [16]. Cell cycle arrest in a panel of DLBCL cells is accompanied by cyclin-dependent kinases Cdk2 decrease and checkpoint dependent kinase Chk2 increase. Caspase activation is demonstrated by the dose-dependent increase in caspase-3, -8 and -9 cleavage and is accompanied by Poly ADP-ribose polymerase (PARP) cleavage [16].

These data are strengthened by recent evidences demonstrating that eosinophils undergo cell cycle arrest and apoptosis following RSV treatment [29]. When treated with RSV, eosinophils from asthmatic individuals show an increase in the protein levels of p53 and p21 and, concurrently, reduced protein levels of Cdk2, cyclin A and cyclin E. Apoptosis was induced through the increase in the apoptotic mediators Bim and Bax and the downregulation of Bcl2 [29]. 

Polydatin (PD) is a natural precursor of RSV. It has been shown to induce cell cycle arrest and apoptosis in MOLT-4 leukemia cells [30]. This occurs through S-phase arrest, decrease of mitochondrial membrane potential and generation of reactive oxygen species. Furthermore, two cell cycle regulatory proteins, cyclin D1 and cyclin B1, were suppressed by PD.

RSV is able to reduce proliferation and induce apoptosis (again, through the caspase-3 activation and Bax up-regulation) of primary effusion lymphoma (PEL) lymphoma cells. The replication of HHV-8 is essential for PEL cells survival. RSV treatment inhibits HHV-8 replication at the same concentrations capable of reducing cell replication and inducing apoptosis [17].

RSV exerts its activity also at the level of mitochondrial membrane. The mitochondrial apoptotic pathway plays a relevant role during cancer cell death through the changes in mitochondrial membrane potential that lead to the subsequent cytochrome c release in different cancer cell models [18,31]. In particular, DLBCL cells treated with RSV dephosphorylate the Akt survival mediator, FOXO1 transcription factor, GSK1 and Bad apoptotic mediator. They undergo changes in mitochondrial membrane potential and eventually to cytochrome c release [19]. The generation of reactive oxygen species (ROS) plays a relevant role during the apoptosis onset. RSV causes ROS release and this seems to be a relevant mechanism of RSV-induced cell death. Interestingly, pretreating DLBCL cells with N-acetyl-cysteine (a ROS scavenger), as well as with anti-oxidant molecules like PEG-catalase or PEG-superoxide dismutase, significantly reduces RSV-induced apoptosis [19].

A few studies were also performed on normal human B lymphocytes. These report a dual effect of RSV on CD19^+^ B lymphocytes: 5 μM of RSV increased significantly the proliferation of CD19^+^ B lymphocytes, while, on the contrary, a concentration of 10 μM inhibited B lymphocyte proliferation and increased caspase-3 activation [32].

### 2.2. The ER Stress

Endoplasmic reticulum (ER) has become a potential target for anticancer therapy since cell death may occur through ER-stress. The classical compensatory response to ER stress is the unfolded protein response (UPR) that reduces protein load in the ER [33]. During a screening of drug-like molecules based on luciferase-reporter gene, RSV, pterostilbene (a natural di-methylated analog of RSV) and piceatannol (another RSV derivative) result in the most effective ER-stress inducers among plant stilbenes [33]. In a model of ER-stress, evaluated on HT1080 fibrosarcoma cells, pterostilbene resulted in the most potent among the three selected plant stilbenes. These three stilbenes were also tested on RCH-ACV and 697 acute lymphoblastic leukemia (ALL) cells. Since autophagy is a mechanism that can partially compensate the ER-stress, the authors tested the three stilbenes in combination with chloroquine, an inhibitor of autophagic decompression. The combination of chloroquine with RSV, piceatannol and pterostilbene induces significant cell death and toxicity that the three stilbenes alone do not induce [33].

The UPR is activated by chemical insult or nutrient deprivation and leads to the activation of stress signaling pathways. The UPR is the major protective and compensatory mechanism enabling stressed cells to survive during ER-stress [22]. The CHOP/GADD153 component (a member of CCAAT/enhancer-binding protein family) is an apoptotic effector protein induced by ER-stress and located downstream of the UPR pathway [34,35]. During RSV-mediated apoptosis of Burkitt lymphoma Raji and Daudi cells, CHOP/GADD153 expression is up-regulated in a dose-dependent fashion and is at least partially responsible for RSV-induced cell death [22].

### 2.3. The Antioxidant and Pro-Oxidant Activities

RSV may exert antioxidant activity at certain concentrations but becomes a pro-oxidant under different conditions. The pro-oxidant activity is considered one of the mechanisms used by RSV to cause tumor cell death. Oxidants have been widely shown to initiate the cellular apoptotic cascade by perturbing the balance between cellular signals for survival and death. 

The pro-oxidant activity of three RSV hydroxylated analogs has been demonstrated on Jurkat T-cell leukemia cells [36]. The oxidative stress, decrease of glutathione levels and loss of superoxide dismutase (MnSOD) activity are all characteristics that accompany Jurkat cells apoptosis. The cytotoxicity may be due to the hydroxylated RSV metabolites that are pro-oxidative and may arise from the parental RSV molecule [36].

ROS are known to induce apoptosis of cancer cells [37,38]. Tumor necrosis factor (TNF)-related apoptosis-inducing ligand (TRAIL) emerged as one of the main players of receptor-mediated apoptosis. TRAIL leads to apoptosis upon ligation to its death receptors DR4 and DR5. Upregulation of DR4 and DR5 increases the sensitivity to TRAIL in various cell lines including KBM-5 (human chronic leukemic cells) [38]. The upregulation of death receptors is mediated by ROS and the involvement of ROS had been demonstrated also in the RSV-mediated apoptosis of DLBCL cell lines [19].

The production of ROS induced by RSV has been also demonstrated in PEL cells [17]. Reduced glutathione (GSH) is the most abundant low molecular weight thiol in human cells. GSH contributes to maintain redox homeostasis within the cell protecting the organelles through the oxidation of the cysteine of its thiol group leading to the formation of oxidized glutathione (GSSG) [23]. The intracellular loss of GSH is an early hallmark in the progression of cell death induced by death receptor-mediated apoptosis [39].

### 2.4. Differentiation and Death Receptor Pathway

Another pathway that RSV has been implicated with is the differentiation of anaplastic large cell lymphoma (ALCL) cells [40]. ALCL is a specific mature B-cell neoplasm bearing CD30 surface marker. RSV treatment is able to induce the expression of differentiation markers like CD2, CD3 and CD8. Furthermore, RSV treatment on SR-786 ALCL-derived cell line upregulates the death receptors CD95/Fas at the surface of the target cells [40].

The involvement of the CD95 signaling cascade had been previously demonstrated by experiments showing that RSV triggers CD95-mediated apoptosis in human promyelocytic leukemia cell line HL-60 [41]. Specifically, RSV treatment up-regulates CD95L expression at the surface of HL-60 cells after 24 hours incubation, while it seems to target specifically CD95^high^ cells, leaving CD95^low^ cells alive. This effect is accompanied by annexin V^+^ cells increase and PARP1 cleavage [41]. Interestingly enough, the same paper also reports that normal human peripheral blood lymphocytes (PBLs) are not significantly affected by the same concentration of RSV. Furthermore, RSV-treated PBLs do not upregulate CD95L, at variance with HL-60 cells. Therefore, the authors conclude that, in normal PBLs, RSV does not induce apoptosis because it does not trigger the CD95-CD95L signaling. 

### 2.5. EBV^+^ Burkitt’s Lymphoma

The anticancer properties of RSV have been also tested on EBV^+^ Burkitt’s lymphoma (BL) cell lines. EBV infection can confer an additional survival potential to malignant B cells, also depending on the different programs of latent gene expression adopted by the virus [24]. The EBV lytic antigen expression is inhibited by RSV at the concentrations corresponding to the respective IC_50_s for growth inhibition of Raji and Akata cells [25]. RSV consistently inhibits viral particle production. The treatment of latently infected BL cells with RSV causes the cell cycle arrest, leading ultimately to apoptosis. The treatment with RSV also inhibits the immediate early as well as the early viral genes at the post-transcriptional level and ultimately the cell proliferation [25].

### 2.6. Immunological Modulation

Immunotherapy is an effective treatment modality for B-cell lymphomas (exploited by the clinical use of anti-CD20 monoclonal antibodies) and the discovery that the lymphoma-associated Ags could be recognized by T cells added an additional treatment opportunity for these tumors [42].

Notably, RSV is able to modulate B lymphocytes immune functions through the upregulation of human leukocyte antigen (HLA) class II proteins. RSV induces the up-regulation of both DR and DM HLA class II molecules in Human Burkitt’s lymphoma cell lines Nalm-6, Ramos and Daudi [26]. Burkitt’s lymphoma seems not to be able to present HLA class I antigen and this contributes to escaping immune recognition from tumor-specific CD8^+^T cells [43]. RSV was used at the concentration of 50 and 100 μM to up-regulate HLA class II.

Acidic cathepsins are responsible for the processing of antigens and class II molecules within endolysosomal compartments of B cells. Antigen processing is a relevant step for B-cell lymphoma recognition by the immune system. Active cathepsins S, B, and D were increased following treatment with 50 μM RSV, consistent with the concomitant HLA class II up-regulation. Furthermore, RSV treatment enhances endocytic recycling of HLA class II proteins in the early endosomal compartments resulting in increased class II presentation and immune activation in the tested B-cell lines [26].

## 3. Cell Cycle Progression as a Target in Lymphomas

Cell cycle kinase activities can be upregulated in cancer either through the overexpression of cyclins and cyclin-dependent kinases (Cdks), or through the inactivation of the Cdk inhibitors [7]. RSV causes cell cycle arrest of several types of cancer cells in G1/S phase and in S phase. The anti-proliferative activity of RSV involves the induction of p21WAF1 and p27KIP1 and downregulation of cyclins D1/D2/E, Cdks 2/4/6, and hyperphosphorylated retinoblastoma protein (pRb) [44,45]. In different models, including lymphoma and leukemia, RSV arrests the cell cycle at the S-phase [9,46,47] or at the G2/M-phase through Cdk7 and p34Cdc2 kinases inhibition [48].

The tumor suppressor p53 is also upregulated by RSV, following BCL6 downregulation, in OCI-Ly8 follicular lymphoma cells [27]. During RSV-induced cell cycle arrest, the phosphatidylinositol 3-kinase (PI3K) signaling and glucose metabolism are inhibited. The treatment with RSV inhibited glycolysis and arrested the cell cycle of OCI-Ly1 and OCI-Ly18 DLBCL cells [20].

The PI3K/Akt pathway plays a recognized oncogenic role at the levels of survival and proliferation of DLBCL cells and tumors and PI3K is frequently activated leading to phosphorylation of downstream substrates like Akt, FKHR, and GSK3 [21].

Interestingly, Cdk1 is inhibited by RSV [28]. Cells overexpressing the oncogene MYC undergo rapid apoptosis when treated with Cdk1 inhibitors. Cdk1 inhibitors lead to survivin (a known Cdk1 target) downregulation and induce a MYC-dependent apoptosis. The treatment with Cdk1 inhibitors is consistently effective also in MYC-dependent mouse lymphoma and hepatoblastoma tumors, suggesting that RSV may be a potential new drug targeting MYC oncogenic pathway [49].

Checkpoints are key regulators of the progression of the cell through the cell cycle. DNA altered due to DNA-damaging agents or UV radiation is detected by sensors that activate checkpoint pathways aimed at arresting the cell cycle in the G1, S or G2 phases. Proteins such as ATM and ATR are DNA-damage sensors and promote cell cycle arrest, DNA repair and, possibly, apoptosis. RSV induces S phase cell cycle arrest in malignant B cells (HS-sultan Burkitt’s lymphoma and IM9 myeloma cells) [50]. This is achieved through the phosphorylation of ATM, checkpoint kinase (Chk) 1 and Chk2 [50].

## 4. Molecular Targets during Apoptosis Onset in Leukemias

RSV has been shown to inhibit the in vitro growth of human acute lymphocytic and non-lymphocytic leukemia cell lines, including HL-60 (promyelocytic leukemia) [3,41], K562 (myeloid leukemia/chronic myelogenous leukemia) [51], and CEM–C7H2 (T-acute lymphocytic leukemia) [52].

**Figure 2:**
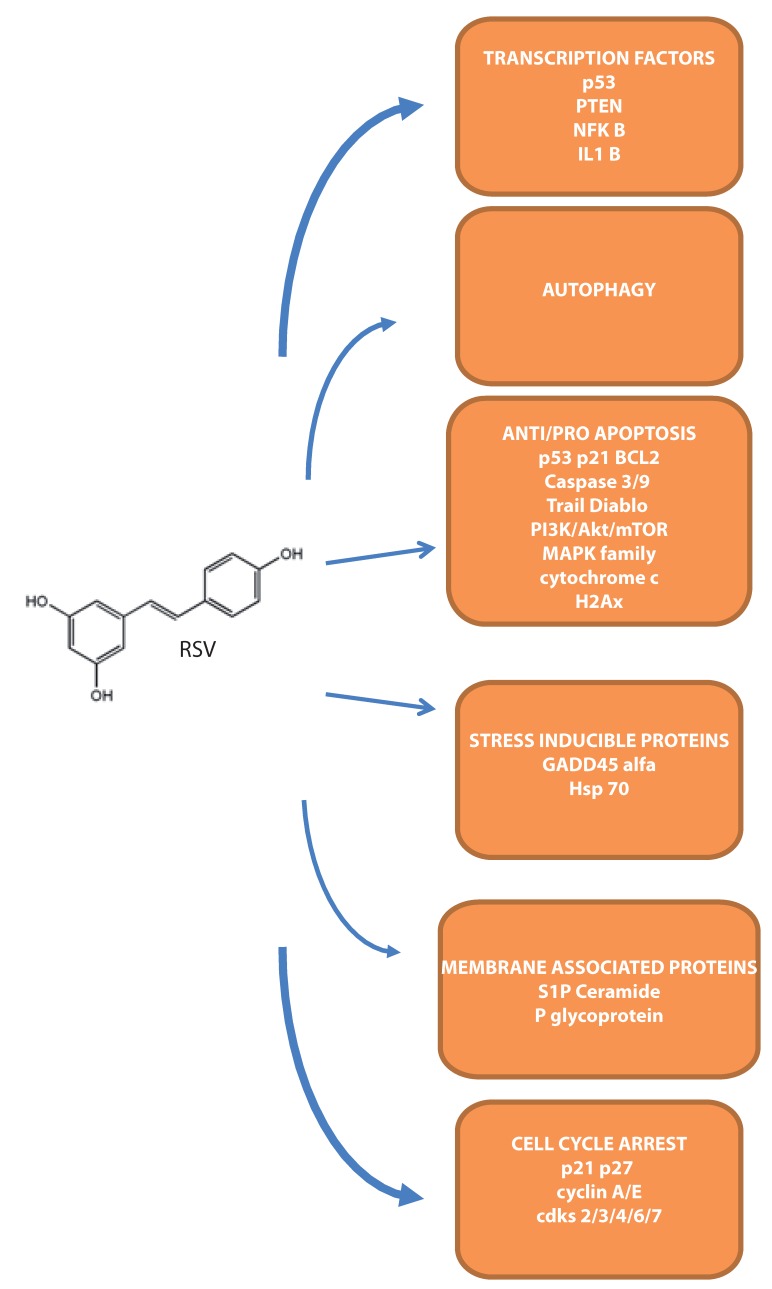
Overview of the main molecular targets demonstrated in leukemia cells.

A number of mechanisms have been proposed for the growth inhibitory effect of RSV on leukemia cells including induction of differentiation, apoptosis, cell cycle arrest and inhibition of DNA synthesis [53]. The antiproliferative effects of RSV involve different pathways, which may be dependent on both concentrations of the drug and characteristics of target cells. RSV exerts its ability to modulate these pathways in the micromolar range of concentrations [54]. The targets are summarized in Figure 2 and Table 2.

### 4.1. Intracellular Apoptotic Mediators 

It is known that the RSV can act as an antioxidant or a pro-oxidant agent depending on the redox status of phenolic hydroxy groups and the electron delocalization across its chemical structure [64].

Some studies have demonstrated a pro-oxidant effect at higher concentrations, while at lower concentrations (5 μM–10 μM) RSV functions as antioxidant. The cellular redox state is a product of intracellular levels of reactive oxygen species (ROS) (such a superoxide (O^2−^) and hydrogen peroxide (H_2_O_2_) and the ability of the cells to maintain a non-toxic constitutive concentration of these reactive species via the antioxidant defense mechanisms. Elevated intracellular levels of H_2_O_2_ trigger apoptotic signals while, when O^2−^predominates, apoptotic signaling is inhibited [65]. Ahmad et al. observed that low RSV concentrations (4–8 μM) inhibited caspase activation as well as DNA fragmentation induced by H_2_O_2_ in human leukemia cells. The inhibitory effects of RSV is not due to its antioxidant activity, but to an increased O^2−^ production that creates a pro-oxidant intracellular environment, nonpermissive for caspases activation and cell death. However, higher doses of RSV induced apoptosis via caspase-3 cascade activation in both normal (60 μM) and leukemic (5–43 μM) hematopoietic cells [64,66].

Several studies show that RSV induces apoptosis by means of different pathways including receptor-mediated or caspase-8-dependent pathway and mitochondrial or caspase-9-dependent pathway [53].

RSV can induce the expression of CD95L and activate CD95-signaling-dependent apoptosis in HL-60 cells [67]. Once triggered, the CD95 receptor can increase caspases activity detected by PARP cleavage that occurs at the onset of apoptosis. Furthermore, Bernhard et al. found that RSV caused arrest in the S-phase prior to Fas-independent apoptosis in CEM-C7H2 ALL cells [52].

However, many kinds of ALL of the B-cell lineage are resistant to CD95-mediated apoptosis [68]. In these cells, RSV causes the depolarization of mitochondrial membranes leading to the activation of caspase-9 which initiates the apoptotic process [55].

RSV inhibits proliferation and induces apoptosis in human leukemia K562 cells through the regulation of cell cycle machinery and activation of mitochondria-mediated caspase-3 dependent apoptotic signaling cascades [56]. 

Several families of protein kinases regulate the complex events that drive the cell cycle and alterations in their activity are frequent in cancer cells. RSV can arrest cancer cells at the G1/S phase inducing p21WAF1 and p27KIP1 and downregulating Cdks 2/4/6. In other cell types, RSV has been reported to arrest the cell cycle at the S-phase as well as at the G2/M-phase, by inhibiting Cdk7 and p34Cdc2 kinases [7].

Ragione et al. demonstrated that RSV causes a complete and reversible cell cycle arrest at the S phase checkpoint. This block was not due to the induction of apoptosis, but to a differentiation towards the myelo-monocytic phenotype. Furthermore, an increase of cyclins A and E was observed along with the accumulation of cell division cycle 2 (CdC2) in the inactive phosphorylated form. These are all elements indicating an arrest of the cell cycle at the S phase checkpoint [67].

Cell cycle arrest is also observed during the RSV-mediated inhibition of cell proliferation of Natural Killer (NK) malignant cells. RSV acts in a time- and dose-dependent fashion by blocking the cell cycle at G0/G1 phase, downregulating CdC2, Cdk2, Cdk3 and inducing apoptosis [61]. Signal transducer and activator of transcription 3 (STAT3) is important for survival and proliferation of malignant NK cells. RSV is able to inhibit phosphorylation and acetylation of STAT3 and expression of anti-apoptotic signals downstream of STAT3, as survivin, Bcl-10 and myeloid cell leukemia 1 (MCL1). RSV activates caspase-3 even though functional p53 is not required for anti-tumor activity against malignant NK cells [61].

Chronic myelogenous leukemia (CML) cells have been investigated as well. RSV induces the apoptosis of K562 cells via phosphorylation of histone H2AX at ser139 in a time- and dose-dependent manner. H2AX belongs to the histone H2A family and is a novel suppressor protein with a role in the regulation of cancer cell apoptosis. RSV triggers the activation of mitogen-activated protein kinase (MAPK) family members p38 and JNK and this is followed by H2AX phosphorilation. [60]. RSV triggers apoptosis in CML cell lines via caspase-3 activation and Bcl2/Bax ratio decrease that probably causes a DNA damage. Indeed, RSV increases gammaH2AX phosphorylation via ATM/ATR kinases.

CML is currently treated with purine analogs, such as fludarabine or cladribine. However, their use bears undesired effects as toxicities, immune-suppression, vomiting or hepatic lesions and some patients are refractory to these drugs. Interestingly, there is an higher apoptosis rate in cells treated with RSV in combination with purine analogs than that caused by a single drug. These results indicate a synergistic effect that might lead to hypothesize lower doses of purine analogs. These data may be especially interesting for older patients for whom there are some limitations to the use of aggressive treatments [69].

The RSV chemopreventive activity is demonstrated also through inhibition of the PI3K/Akt/mTOR signaling cascades [70], which plays a vital role in oncogenic transformation and cancer progression. Indeed, the phosphorylation of Akt and mTOR decreases after treatment with RSV in a time- and concentration-dependent manner, while p38-MAPK signal pathways is activated by phosphorylation of p38 in leukemia cells [58,59].

RSV induces a G0/G1 cell cycle arrest via upregulating CDK-inhibitors p21 and p27 and downregulating cyclin A and cyclin D1. RSV inhibits the phosphorylation of Akt, mTOR in a time-dependent manner and increases the phosphorylation of p38, together to caspase-3 cleavage and pro-apoptotic Bim, Bad and Bax up-regulation. Anti-apoptotic Bcl-2 and Mcl-1 result downregulated consistent with observed cell death. RSV not only induces apoptosis in T-cell acute lymphoblastic leukemia (T-ALL) cells, but also autophagy that negatively modulates apoptosis. Indeed, blocking autophagy with the specific inhibitor 3-methyladenine (3-MA) increases RSV-induced apoptosis, suggesting that RSV-induced autophagy might contribute to chemo-resistance in T-ALL cells [58].

The CEM-C7H2 T-ALL cell line constitutively overexpresses Bcl-2. RSV causes cell death via a novel mitochondrial signaling pathway, potentially antagonized by Bcl-2. RSV stimulation of these cells produces ROS, mitochondrial membrane potential decrease and activation of caspases 9-2-3-6 without inducing cytocrome c release. On the contrary, all these processes are significantly reduced by overexpression of Bcl-2 that neutralizes the mitochondrial membrane potential decrease and the ROS production [71].

### 4.2. Transcription Factors Modulated by RSV

RSV can cause apoptosis through p53-dependent and p53-independent pathways. Several studies show the activation of the p53-dependent pathway demonstrating that chronic administration of RSV at a sub-apoptotic dose results in senescent-like growth arrest in different carcinoma cell lines [72]. This effect is due to increased ROS generation, ataxia telangiectasia mutated kinase (ATM) and p53 activation (via p38-MAPK-mediated p53 phosphorylation at serine 15, induction of p21, and subsequent induction of senescence).

RSV is also reported to induce apoptosis via activation of both intrinsic (mitochondria-mediated) and extrinsic (death receptor-mediated) pathways [73]. RSV increases p53-mediated expression of pro-apoptotic proteins (e.g., Bax, Bak, Bim, PUMA, Noxa, etc.) and the release of mitochondria-specific proteins (e.g., cytochrome c, Smac/DIABLO, etc.) to the cytosol, thus triggering suppression of inhibitors of apoptosis proteins (e.g., Bcl2, Bcl-XL, survivin, XIAP, etc.) and caspase activation in several cancers [74].

In a model of ALL, RSV affects the post-translational modifications of p53. RSV increases p53 phosphorylation at serine15 residue and hyper-activates Phosphatase and tensin homolog (PTEN), a tumor suppressor that negatively regulates the protein kinase B (Akt/PKB) signaling pathways [75].

It was demonstrated that the nuclear transcription factor NF-kB is a regulator of the immune system and triggers the expression of various cytokines, as interleukin 1 beta(IL1β). RSV is able to suppress the production of IL1β and NF-kB in a dose-dependent manner in the acute myeloid leukemia (AML) cell lines OCI/AML3 and OCIM2. RSV leads to S-phase arrest of the cell cycle, eventually inducing apoptotic cell death through caspase-3 pathway and subsequent PARP cleavage [47].

### 4.3. Stress Inducible Proteins Involved in RSV–Mediated Cell Death

It was demonstrated that human promyelocitic leukemia cells HL-60 undergo proliferation reduction after RSV treatment. RSV causes the G0/G1 phase arrest and leads to smaller and less refractive cells, showing a typical pattern of nuclear fragmentation (one of the hallmarks of apoptosis). The apoptotic onset is accompanied by Bcl-2 expression decrease, Bax increase and caspase-3 activation. GADD45α is a protein with a role of tumor suppressor involved in regulation of many cellular functions as DNA repair, cell cycle control, senescence and genotoxic stress. Moreover, induction of GADD45α expression is an essential step for mediating apoptotic signaling pathways triggered by multiple chemotherapeutic drugs. RSV also increases protein level of GADD45α in a time- and dose-dependent fashion [57].

Several studies have demonstrated that heat shock proteins Hsp70 and Hsp90 are contributors to oncogenesis, and that overexpression of the heat-shock protein 70 is associated with resistance in chronic myeloid leukemia [76,77]. In normal cells Hsp genes are transcribed by the heat shock factor (HSF) family that is activated by heat or other forms of stress [76]. When the coordination of HSF1 activation, stress response and post recovery deactivation mechanism is not well orchestrated, the Hsps become highly overexpressed. This may render the cells anti-apoptotic and eventually leads to malignancy [76]. Interestingly, RSV has the property of downregulating Hsp70 both at the mRNA and protein levels in K562 cells in a time- and dose-dependent manner [5,63].

Heat-shock proteins inhibitors became the also the targets for pharmacological research in order to translate them into the clinic. Among these, 17-Allylamino-demethoxygeldanamycin (17AAG) is one of the most promising for the therapy of CML [76,78]. 17AAG effectively targets Hsp90 but, unfortunately, its efficacy is limited by the fact that Hsp70 results simultaneously induced [79,80]. This characteristic limits the therapeutic potential of 17AAG. Thanks to its ability to modulate the heat shock response, RSV was used in combination with 17AAG on K562 cells and improved the pro-apoptotic efficacy of this heat-shock protein inhibitor [63].

### 4.4. The Involvement of Membrane-Associated Proteins

Multiple studies have demonstrated that sphingolipids and their metabolites are critical players in many biological processes important for health and disease. Specifically, ceramide, sphingosine and sphingosine 1-phosphate (S1P) represent interconnected effector molecules with opposite functions. Indeed, S1P promotes cell survival and proliferation, while ceramide and sphingosine induce cell growth arrest and apoptosis. Hence, the balance between ceramide and S1P is important to determine the faith of cancer cells (growth inhibition versus survival) [81]. Crucial regulators of this balance are Sphingosine Kinase (SphKs) which catalyze the phosphorylation of sphingosine to S1P at the expense of its pro-apoptotic precursors sphingosine and ceramide. Two distinct SphK isoforms have been identified: SphK1 and SphK2. SphK1 is overexpressed in various types of cancers including acute leukemia and upregulation of SphK1 has been associated with tumor angiogenesis and resistance to radiation and chemotherapy. SphK1 is mainly a cytosolic enzyme and when migrates to the plasma membrane it generates S1P [81]. RSV may regulate leukemia cell proliferation and induce apoptosis via its modulation of SphK1/S1P signaling restoring the balance between S1P and ceramide [62].

Multidrug resistance is often associated with the overexpression of P-glycoprotein (P-gp) in leukemic cell membranes [82,83,84]. P-gp is a transmembrane pump that reduces caspase-dependent cell death not only by counteracting intracellular accumulation and toxicity of anti-tumor drugs but also by inhibiting the activation of caspases. The expression of P-gp can be modulated by PI3K/Akt pathways which play a role in oncogenic transformation and cancer progression [84].

It was demonstrated that RSV is capable of downregulating P-gp expression at mRNA and protein levels. RSV-mediated downregulation is time- and concentration–dependent in K562 cells and occurs through the inhibition of PI3K/Akt pathway [59].

## 5. Conclusions 

The cellular targets treated in this review represent a broad spectrum of cellular activities and mechanisms of action that are affected by RSV. Lymphoma and leukemia might benefit from a treatment or pre-treatment with RSV during a combined therapy. The pro-apoptotic and pro-oxidant effects represent a useful tool to sensitize cancer cells to a subsequent chemotherapy. This is true as well for the enhancement of TRAIL activity. 

These applications are especially useful since RSV is a molecule without known severe toxicities. Furthermore, the observed downregulating effects on P-glycoprotein can be used to hypothesize a treatment with RSV in case of resistance insurgence due to drug extrusion. The range of micromolar concentrations can be considered a good starting point for an in vitro combined therapy approach. 

Since lymphoma and leukemia arise from immature or defective cells of the immune system, the evidence that RSV increases HLA class II expression and antigen processing in some lymphoma models represents a very attractive approach for immunotherapy strategies.

The sad side is that RSV clinical utility has been hampered by the low bioavailability of the parental molecule during in vivo experimental trials, even though animal data are promising. Glucuronide- and sulfonate-conjugates are rapidly produced by the intestine and liver metabolism. The result is that actual RSV plasma concentration is below the concentration demonstrated for in vitro experiments to show pharmacological activity. These concepts have been well summarized by a panel of experts who designed the guidelines for a future use of RSV in a translational perspective [85]. More trials are recommended, including human clinical trials. These clinical studies should provide the role and distribution of RSV metabolites in the blood, encompassing the hematological tumors. The low toxicities demonstrated in several settings should become a positive starting point for the use of these natural molecules in combination with the most up-to-date anticancer drugs. For these reasons, the availability of standardized RSV, glururonide- and sulfonate-metabolites formulations are key to develop these kinds of trials.

Collectively, the evidence discussed here encompasses a wide range of cellular and molecular targets that should be combined and exploited in order to improve and integrate the current therapeutic approaches for lymphoma and leukemia.

## Figures and Tables

**Table 1:** Summary of the lymphoma molecular targets cited in the text.

Pathway Affected	Cell Type	Concentration Ranges	References
*Apoptosis*			
Caspase 3, 8, 9	NU-DUL-1; OCI-Ly8; U2932; SUDHL-4; DB; TMD8	24–32 μM; 20 μM	[16,17]
PARP	SUDHL-4; NU-DUL-1	24–32 μM	[16]
Bax/Bcl2	SUDHL-4; NU-DUL-1	24–32 μM; 20 μM	[16,17]
Cytochrome c	SUDHL-4; HBL-1	25 and 50 μM	[18,19]
*Cell survival*			
PI3K/Akt	SUDHL-4; SUDHL-5	25 and 50 μM	[20,21]
*ER-stress*			
CHOP/GADD153	Raji; Daudi	10–200 μM	[22]
*Pro-oxidant activity*			
ROS	BCBL-1; BC-1; P3HR1; BJAB	20 μM	[17]
GSH depletion	U-937	50 μM	[23]
*Death receptor pathway*			
DR5	SUDHL-4; HBL-1	25 and 50 μM	[19]
*EBV infection*			
EBV lytic antigens	Raji; Akata	20–300 μM	[24,25]
*Antigen presentation*			
DR and DM HLA class II	Nalm-6, Ramos, Daudi	50 μM	[26]
Active cathepsins S, B, and D	Nalm-6, Ramos, Daudi	50 μM	[26]
*Cell cycle*			
S-phase	L-428	25 and 50 μM	[9]
p53; Bcl6; PI3K	OCI-Ly1; OCI-Ly18	25 μM	[20,27]
Cdk1	Mouse lymphoma	1–150 μM	[28]

**Table 2:** Summary of the leukemia molecular targets cited in the text.

Pathway Affected	Cell Type	Concentration Ranges	References
*Apoptosis*			
CD95/caspase-3	HL-60; K562	1–100 μM	[55,56]
Caspase-9	SEM; RS4:11; MV4:11; REH; NALM-6; CEM; JURKAT; HL-60	50 μM	[55]
Cytochrome c	CEM; Molt-4	25 and 50 μM	[18,19]
PARP	OCI/AML3; OCIM2	5–75 μM	[47]
GADD45α	HL-60	12.5–200 μM	[57]
*Autophagy*			
MDC (autophagosomes)/phagophore/LC3	Molt-4; Jurkat; CEM	25–250 μM	[58]
*Cell survival*			
NF-kB	OCI/AML3; OCIM2	5–75 μM	[47]
IL-1	OCI/AML3; OCIM2	5–75 μM	[47]
PI3K/Akt	Molt-4; Jurkat; CEM	25–250 μM; 10 μM	[58,59]
*Cell proliferation*			
p38; JNK (MAPK family)	K562	20–100 μM	[60]
*Cell cycle*			
cyclins 2/6	malignant NK	12.5, 25 and 50 μM	[61]
*Plasma membrane associated targets*			
Sphingosine kinases/sphingosine 1P	K562	20 and 40 μM	[62]
P-glycoprotein	K562	10 μM	[59]
*Heat shock proteins*			
HSP70/HSP90	K562	20–100 μM; 40 μM	[5,63]
